# Supplementary material for: Leptospirosis in the Caribbean Region between 2000 and 2022: A scoping review of morbidity and mortality
Source: PLoS Negl Trop Dis. 2026 Jan 5;20(1):e0013595. doi: 10.1371/journal.pntd.0013595 (PMC12782409; doi:10.1371/journal.pntd.0013595)
Supplement: S3 Table — (DOCX) [file pntd.0013595.s003.docx]

**Supporting Table 3. The complete list of the 48 publications (no time restriction) reporting leptospirosis cases or seroprevalence in at least one of the 27 CRICTs and the period in which the study was conducted.**

| **First author, year** | **Study years** | **Anguilla** | **Antigua and Barbuda** | **Barbados** | **British Virgin Islands** | **Cayman Islands** | **Cuba** | **Dominica** | **Dominican Republic** | **Grenada** | **Guadeloupe** | **Haiti** | **Jamaica** | **Martinique** | **Montserrat** | **Puerto Rico** | **Saint Kitts and Nevis** | **Saint Lucia** | **Saint Vincent and the Grenadines** | **Trinidad and Tobago** | **U.S. Virgin Islands** |
| --- | --- | --- | --- | --- | --- | --- | --- | --- | --- | --- | --- | --- | --- | --- | --- | --- | --- | --- | --- | --- | --- |
| Adesiyun, 2010 | 2006 |  |  |  |  |  |  |  |  |  |  |  |  |  |  |  |  |  |  | x |  |
| Adesiyun, 2011 | 1997-2005 | x | x |  | x | x |  | x |  | x |  |  | x |  |  |  | x | x | x | x |  |
| Artus, 2022 | 2019 |  |  |  |  |  |  |  |  |  |  |  |  |  |  |  |  |  |  |  | x |
| Batchelor, 2012 | 1992-2007 |  |  |  |  |  |  |  |  |  |  |  |  |  |  |  |  |  |  |  |  |
| Bennet, 1991 | 1979-1986, |  |  | x |  |  |  |  |  |  |  |  |  |  |  |  |  |  |  |  |  |
| Briskin, 2019 | 2015 |  |  |  |  |  |  |  |  |  |  |  |  |  |  | x |  |  |  |  |  |
| Bruce, 2005 | 1996-1997 |  |  |  |  |  |  |  |  |  |  |  |  |  |  | x |  |  |  |  |  |
| Cassadou, 2016 | 2011 |  |  |  |  |  |  |  |  |  | x |  |  | x |  |  |  |  |  |  |  |
| Chery, 2020 | 2010-2017 |  |  |  |  |  |  |  |  |  |  |  |  |  |  |  |  | x |  |  |  |
| Damude, 1979*^1^ | 1968-1974 |  |  | x |  |  |  |  |  |  |  |  |  |  |  |  |  |  |  |  |  |
| Damude, 1979*^2a^ | 1975-1977 |  |  | x |  |  |  |  |  |  |  |  |  |  |  |  |  |  |  |  |  |
| Damude, 1979*2^b^ | 1975-1977 |  |  | x |  |  |  |  |  |  |  |  |  |  |  |  |  |  |  |  |  |
| Damude, 1979*2^c^ | 1975-1977 |  |  | x |  |  |  |  |  |  |  |  |  |  |  |  |  |  |  |  |  |
| Edwards, 1990 | 1983-1986 |  |  | x |  |  |  |  |  |  |  |  |  |  |  |  |  |  |  |  |  |
| Everard, 1985 | 1977-1978 |  |  |  |  |  |  |  |  |  |  |  |  |  |  |  |  |  |  | x |  |
| Everard, 1987 | 1977-1982 |  |  |  |  |  |  |  |  |  |  |  |  |  |  |  |  |  |  | x |  |
| Everard, 1989 | 1980-1983 |  |  |  |  |  |  |  |  |  |  |  |  |  |  |  |  |  |  | x |  |
| Everard, 1979*^1^ | 1975-1978 |  |  |  |  |  |  |  |  | x |  |  |  |  |  |  |  |  |  |  |  |
| Everard, 1979*^2^ | 1975-1978 |  |  |  |  |  |  |  |  | x |  |  |  |  |  |  |  |  |  |  |  |
| Everard, 1984 | 1979-1982 |  |  | x |  |  |  |  |  |  |  |  |  |  |  |  |  |  |  |  |  |
| Everard, 1992 | 1979-1982 |  |  | x |  |  |  |  |  |  |  |  |  |  |  |  |  |  |  |  |  |
| Everard, 1995 | 1979-1991 |  |  | x |  |  |  |  |  |  |  |  |  |  |  |  |  |  |  |  |  |
| Gale, 1990 | 1984-1988 |  |  | x |  |  |  |  |  |  |  |  |  |  |  |  |  |  |  |  |  |
| Gentilini, 1964*^1^ | 1959 |  |  |  |  |  |  |  |  |  |  | x |  |  |  |  |  |  |  |  |  |
| Gentilini, 1964*^2^ | 1959 |  |  |  |  |  |  |  |  |  |  | x |  |  |  |  |  |  |  |  |  |
| Golden, 2014 | 2007-2008 |  |  |  |  |  | x |  |  |  |  |  |  |  |  |  |  |  |  |  |  |
| Gonzalez, 1976 | 1973 |  |  |  |  |  | x |  |  |  |  |  |  |  |  |  |  |  |  |  |  |
| Grant, 1964*^1^ | 1953-1963 |  |  |  |  |  |  |  |  |  |  |  | x |  |  |  |  |  |  |  |  |
| Grant, 1964*^2^ | 1953-1963 |  |  |  |  |  |  |  |  |  |  |  | x |  |  |  |  |  |  |  |  |
| Herman-Storck, 2005 | 1994-2001 |  |  |  |  |  |  |  |  |  | x |  |  |  |  |  |  |  |  |  |  |
| Herman-Storck, 2008 | 2003-2004 |  |  |  |  |  |  |  |  |  | x |  |  |  |  |  |  |  |  |  |  |
| Hiatt, 1976 | 1974 |  |  |  |  |  |  |  |  |  |  |  |  |  |  | x |  |  |  |  |  |
| James, 2013 | 2010-2011 |  |  |  |  |  |  |  |  |  |  |  |  |  |  |  |  |  |  | x |  |
| Jones, 2024 | 2022 |  |  |  |  |  |  |  |  |  |  |  |  |  |  | x |  |  |  |  |  |
| Levett, 2000 | 1995-1997 |  |  | x |  |  |  |  |  |  |  |  |  |  |  |  |  |  |  |  |  |
| Lhomme, 1996 | 1987-1992 |  |  |  |  |  |  |  |  |  |  |  |  | x |  |  |  |  |  |  |  |
| Lindo, 2013 | 2007-2008 |  |  |  |  |  |  |  |  |  |  |  | x |  |  |  |  |  |  |  |  |
| Mohan 2009 | 1996-2007 |  |  |  |  |  |  |  |  |  |  |  |  |  |  |  |  |  |  | x |  |
| Nilles, 2024 | 2021 |  |  |  |  |  |  |  | x |  |  |  |  |  |  |  |  |  |  |  |  |
| Perez, 1998 | 1987-1993 |  |  |  |  |  | x |  |  |  |  |  |  |  |  |  |  |  |  |  |  |
| Sanchez, 1993 | 1986-1990 |  |  |  |  |  | x |  |  |  |  |  |  |  |  |  |  |  |  |  |  |
| Sanders, 1999 | 1996 |  |  |  |  |  |  |  |  |  |  |  |  |  |  | x |  |  |  |  |  |
| Sharp, 2016 | 2010 |  |  |  |  |  |  |  |  |  |  |  |  |  |  | x |  |  |  |  |  |
| Strobel, 1992 | 1989 |  |  |  |  |  |  |  |  |  | x |  |  |  |  |  |  |  |  |  |  |
| Suarez-Hernandez, 1999 | 1982-1995 |  |  |  |  |  | x |  |  |  |  |  |  |  |  |  |  |  |  |  |  |
| Suarez-Hernandez, 2001 | 1998 |  |  |  |  |  | x |  |  |  |  |  |  |  |  |  |  |  |  |  |  |
| Suarez-Hernandez. 1995 | 1984-1988 |  |  |  |  |  | x |  |  |  |  |  |  |  |  |  |  |  |  |  |  |
| Villafranca, 2002 | 1996-1998 |  |  |  |  |  | x |  |  |  |  |  |  |  |  |  |  |  |  |  |  |
| Wood, 2014 | 2009-2011 |  | x |  |  |  |  | x |  | x |  |  | x |  | x |  | x | x | x |  |  |
