# Supplementary material for: Leptospirosis in the Caribbean Region between 2000 and 2022: A scoping review of morbidity and mortality
Source: PLoS Negl Trop Dis. 2026 Jan 5;20(1):e0013595. doi: 10.1371/journal.pntd.0013595 (PMC12782409; doi:10.1371/journal.pntd.0013595)
Supplement: S5 Table — (DOCX) [file pntd.0013595.s005.docx]

**Supporting Table** **5. Summary of characteristics of publications based on routine surveillance-based studies.**

| First author, year | Study aims | | | | | | Study design | Diagnostic test | | Ref |
| --- | --- | --- | --- | --- | --- | --- | --- | --- | --- | --- |
|  | Motivated by an extreme weather event? | Estimate Incidence | Identify risk factors | Lab confirmed/ suspected cases | Impact of public health intervention | Describe changes through time | Reporting Site | ELISA | MAT |  |
| Golden, 2014 | Yes |  |  |  | x |  | Mixed | NR | NR | (30) |
| Herman-Storck, 2008 | No | x | x |  |  | x | Hospital | x | x | (26) |
| Cassadou, 2015 | No | x |  |  |  |  | Mixed | x | x | (24) |
| Batchelor, 2012 | No | x | x |  |  |  | Mixed | x | x | (31) |
| Mohan, 2009 | No | x | x |  |  | x | Mixed | x |  | (32) |
| Sharp, 2016 | No |  |  | x |  |  | Mixed | x | x | (27) |
| Jones, 2024 | Yes |  | x | x |  |  | Mixed | x |  | (28) |
| Herman-Storck, 2005 | No |  | x | x |  |  | Hospital |  | x | (25) |
| Chery, 2020 | Yes | x | x | x |  |  | Mixed | x |  | (29) |
