# Supplementary material for: Leptospirosis in the Caribbean Region between 2000 and 2022: A scoping review of morbidity and mortality
Source: PLoS Negl Trop Dis. 2026 Jan 5;20(1):e0013595. doi: 10.1371/journal.pntd.0013595 (PMC12782409; doi:10.1371/journal.pntd.0013595)
Supplement: S6 Table — (DOCX) [file pntd.0013595.s006.docx]

**Supporting Table 6. Summary of characteristics of publications based on seroprevalence studies.**

| First author, year | Study aims | | | | Study design | | | Diagnostic test | | Ref |
| --- | --- | --- | --- | --- | --- | --- | --- | --- | --- | --- |
|  | Motivated by an extreme weather event? | Identify risk factors | Risk group prevalence | Lab confirmed/ suspected cases | Sampling design | Recruitment site | Household setting | ELISA | MAT |  |
| Wood, 2014 | No |  | x |  | NR | NR | NR | X |  | (23) |
| Nilles, 2021 | No | x |  |  | Three-stage, hierarchical random sampling | Community | Mixed |  | X | (39) |
| Lindo, 2013 | No |  |  | x | Random | Mixed | Mixed | X |  | (38) |
| James, 2013 | No | x | x |  | Convenience | Community | Mixed | X | X | (35) |
| Adesiyun, 2010 | No |  | x |  | Convenience | Community | Rural | X |  | (36) |
| Artus, 2022 | Yes | x |  |  | A stratified, random two-stage cluster sampling design | Community | Mixed |  | X | (37) |
| Briskin, 2019 | Yes | x |  |  | Convenience | Community | Urban |  | X | (34) |
