# Supplementary material for: Leptospirosis in the Caribbean Region between 2000 and 2022: A scoping review of morbidity and mortality
Source: PLoS Negl Trop Dis. 2026 Jan 5;20(1):e0013595. doi: 10.1371/journal.pntd.0013595 (PMC12782409; doi:10.1371/journal.pntd.0013595)
Supplement: S9 Table — (DOCX) [file pntd.0013595.s009.docx]

**Supporting Table 9. Serovars included in the MAT panel by country/territory and publication.**

| **Species** | **Serogroup** | **Primary reacting serovar** | **Strain** | **Dominican Republic** | **Guadeloupe** | | **Puerto Rico** | | **Trinidad and Tobago** | **US Virgin Islands** |
| --- | --- | --- | --- | --- | --- | --- | --- | --- | --- | --- |
|  |  |  |  | **Nilles, 2021** | **Hermann-Storck, 2008** | **Hermann-Storck, 2005** | **Sharp, 2016** | **Briskin, 2019** | **James, 2013** | **Artus, 2020** |
| *L. biflexa* | Andaman | Andaman | Ch 11 |  |  |  |  |  | X |  |
| *L. interrogans* | Australis | Bratislava | Jez-Bratislava | X | X | X | X | X | X | X |
| *L. interrogans* | Australis | Australis | Ballico | X | X | X |  |  | X | X |
| *L. interrogans* | Autumnalis | Autumnalis | Akiyami A | X |  | X | X | X |  | X |
| *L. kirschneri* | Autuminalis | Bim | 1051 |  |  |  |  |  | X |  |
| *L. interrogans* | Autumnalis | Rachmati | Rachmat |  |  |  |  |  | X |  |
| *L. borgpetersenii* | Ballum | Ballum | Mus 127 | X | X | X |  |  | X | X |
| *L. borgpetersenii* | Ballum | Arborea | Arborea |  | X | X |  |  |  |  |
| *L. borgpetersenii* | Ballum | Castellonis | Castellon 3 |  |  |  |  | X |  |  |
| *L. borgpetersenii* | Ballum | Not determined | M2 isolate |  |  |  |  | X |  |  |
| *L. interrogans* | Bataviae | Bataviae | Swart |  |  |  |  |  | X |  |
| *L. interrogans* | Bataviae | Bataviae | Van Tienen | X |  | X | X | X |  | X |
| *L. interrogans* | Canicola | Canicola | Ruebush | X | X | X | X |  |  | X |
| *L. interrogans* | Canicola | Canicola | H. Ultrecht IV |  |  |  |  | X | X |  |
| *L. weilii* | Celledoni | Celledoni | Celledoni | X |  |  |  |  |  | X |
| *L. kirschneri* | Cynopteri | Cynopteri | 3522 C | X | X | X | X | X | X | X |
| *L. interrogans* | Djasiman | Djasiman | Djasiman | X |  |  | X |  |  | X |
| *L. kirschneri* | Grippotyphosa | Grippotyphosa | Duyster |  |  |  |  |  | X |  |
| *L. kirschneri* | Grippotyphosa | Grippotyphosa | Moskva V | X |  | X |  |  |  |  |
| Not provided | Grippotyphosa | Grippotyphosa | Mandemakers |  |  |  |  |  | X |  |
| Not provided | Grippotyphosa | Grippotyphosa | no strain name |  |  |  |  |  |  | X |
| *L. santarosai* | Hebdomadis | Borincana | HS 622 | X |  | X |  |  |  | X |
| *L. interrogans* | Hebdomadis | Hebdomadis | Hebdomadis |  |  |  |  | X | X |  |
| *L. interrogans* | Icterohaemorrhagiae | Mankarso | Mankarso | X | X | X | X |  | X | X |
| *L. interrogans* | Icterohaemorrhagiae | Icterohaemorrhagiae | RGA | X | X | X | X | X |  | X |
| *L. interrogans* | Icterohaemorrhagiae | Copenhageni | Fiocruz M20 |  |  | X | X | X |  |  |
| *L. interrogans* | Icterohaemorrhagiae | Copenhageni | Fiocruz L1-130 |  |  | X | X | X |  |  |
| *L. interrogans* | Icterohaemorrhagiae | Icterohaemorrhagiae | Kantorowic |  |  |  |  |  | X |  |
| Not provided | Icterohaemorrhagiae | Copenhageni | Wijnberg |  |  |  |  |  | X |  |
| *L. borgpetersenii* | Javanica | Javanica | Veldrat Bataviae 46 | X |  | X |  |  |  | X |
| *L. weilii* | Javanica | Coxi | Cox |  |  |  |  | X |  |  |
| *L. noguchii* | Louisiana | Louisiana | LSU 1945 |  |  |  |  | X |  |  |
| *L. alexanderi* | Manhao | Manhao 3 | L 60 |  |  |  |  | X |  |  |
| *L. santarosai* | Mini | Georgia | LT 117 | X |  |  | X |  |  | X |
| *L. borgpetersenii* | Mini | Mini | Sari |  | ? |  |  | X |  |  |
| *L. noguchii* | Panama | Cristobali | 1996 K |  |  | X |  |  |  |  |
| *L. noguchii* | Panama | Panama | CZ 214 |  |  | X |  | X | X |  |
| *Unknown/ unclear* | Panama | Mangus | TRVL/CAREC 137774 |  |  | X |  |  |  |  |
| *L. interrogans* | Pomona | Pomona | Pomona | X |  | X | X | X | X | X |
| *L. noguchii* | Pomona | Proechimys | 1161 U |  |  |  |  |  | X |  |
| *L. santarosai* | Pyrogenes | Alexi | HS 616 | X |  |  |  |  |  | X |
| *L. interrogans* | Pyrogenes | Pyrogenes | Salinem | X |  | X | X | X | X | X |
| *L. interrogans* | Sejroe | Hardjo | Hardjoprajitno |  |  |  |  |  | X |  |
| *L.borgpetersenii* | Sejroe | Saxkoebing | Mus 24 |  |  |  |  |  | X |  |
| *L. borgpetersenii* | Sejroe | Sejroe | M 84 |  |  |  |  |  | X |  |
| *L. interrogans* | Sejroe | Wolffi | 3705 | X | X | X |  | X |  | X |
| *L. biflexa* | Semaranga | Patoc | Patoc I |  |  |  |  |  | X |  |
| *L. meyeri* | Semaranga | Semaranga | Veldrat Sem 173 |  |  |  |  |  | X |  |
| *L. santarosai* | Shermani | Shermani | 1342 K |  |  |  |  | X | X |  |
| *L. borgpetersenii* | Tarassovi | Tarassovi | Perepelitsin | X | X | X |  | X | X | X |
| *L. kmetyi* | Tarassovi | Malaysia | Bejo-Iso9 |  |  |  |  | X |  |  |
