# Supplementary material for: Leptospirosis in the Caribbean Region between 2000 and 2022: A scoping review of morbidity and mortality
Source: PLoS Negl Trop Dis. 2026 Jan 5;20(1):e0013595. doi: 10.1371/journal.pntd.0013595 (PMC12782409; doi:10.1371/journal.pntd.0013595)
Supplement: Attachment — Submitted filename: Response to Reviewers.docx [file pntd.0013595.s011.docx]

**Leptospirosis in the Caribbean Region between 2000 and 2022 – a Scoping Review of Morbidity and Mortality**

**Response to reviewers**

**Methods**

**Reviewer #2:**

The objective is clearly articulated with the study's purpose, but does not have and testable hypothesis due to the study design (Scoping review).
This design is appropriate for their objective: "To describe geographical and temporal distribution of leptospirosis epidemiology in Caribbean Island 23 Countries and Territories (CRICTs) and identify patterns and gaps in knowledge"
Definition of study population and statistical analysis does not apply to the study (Scoping review)
Ethical considerations are appropriate for the study type (Scoping review)

**Authors:** We greatly appreciate the time and expertise invested in helping us strengthen this work.

**Reviewer #3:**

The article has the aim to describe geographical and temporal distribution of leptospirosis epidemiology in Caribbean Island Countries and Territories (CRICTs) and identify patterns and gaps in knowledge.
The paper conducted a scoping review of studies published between 2000-2022 to provide an updated overview of leptospirosis epidemiology across CRICTs.
The objectives, methodology and data analysis are suitable for the study’s aim.

However, in the section of data analysis, Line 169-175: the authors described that the aims reported by publications were categorised into 6 groups but the detail are provided only 5 groups.

**Authors:** We sincerely thank the reviewers for their thorough evaluation of our manuscript and for the thoughtful, constructive comments provided.

Regarding the number of categories, the correct is *five groups* and it has been corrected in the main text.

**Reviewer #4:**

The objectives are clearly described, and the study uses the proper methodology by following the PRISM-ScR checklist. In the Search Strategy (Line 129-130), this sentence "In 130 each database, the search term applied was a combination of ‘Leptospirosis’ AND ‘Country/territory’." is not consistent with the Supporting Table 2. It should be "Caribbean", shouldn't it?

**Authors:** Thank you for your time, effort, and constructive suggestion in this paper. Although our study is focused on the Caribbean region, most of the studies were conducted at the country/territory level, if the search was conducted using ‘Caribbean’ instead of each ‘country/territory’, it would not have retrieved many of the relevant studies. Instead, when searching for studies conducted specifically in each of the countries/territories of interest, the final search retrieved publications that provided a more comprehensive characterisation of available data from the region. No changes were made.

**Results**

**Reviewer #2**:

The analysis matches the analysis plan, a description of the evidence.
Results are clearly and completely presented. However, the authors include result interpretation and make judgments within the results section.
The figures are (Tables, Images) of sufficient quality for clarity.

**Authors:** Sentences with results interpretation and judgments were removed from the results section.

**Reviewer #3:**

The results are well presented. Tables and figures are appropriate and clear presentation. Titles, legends, columns and rows labels are mostly correct and clear.
Some minor comments on result sections are as follows:

Line 222: Please check the percentage representing 3 routine surveillance-based studies mentioned being motivated by the occurrence of a specific extreme weather event.

**Authors:** The percentage was corrected and it now reads: “*Three routine surveillance-based studies mentioned being motivated by the occurrence of a specific extreme weather event (33.3%)* (…)”.

Line 254-256: Please check the reference for this data.

**Authors:** The reference (i.e., Wood et al, 2014) has been confirmed.

In this short report, a serosurvey was conducted in 10 English-speaking Caribbean countries to examine the seroprevalence of seven zoonotic diseases. Details regarding recruitment site (e.g., hospitals or clinic or communities), setting (e.g., urban or rural), sampling design (random or census or convenience sampling) and demographic distribution (e.g., age) were not provided.

Line 264: Please check the correction of data in the main text and the S6 table mentioned about 5 seroprevalence studies investigated risk factors.

**Authors:** Thank you for pointing this inconsistency between the main text and the *S6 Table*. The correct number of seroprevalence studies reporting risk factors is four (Nilles et al, 2022; Artus et al, 2022; James et al, 2013; and Briskin et al, 2019). The numbers have been corrected in the main text, and in the *S6 Table*.

Line 273-275: In the sentence “…leptospirosis seroprevalence varied considerably across the CR, ranging from 4.1% in the U.S. Virgin Islands (2019) (28) to 26.9% in Trinidad and Tobago (2010–2011) (32) (Table 2).”
Please check the reference for this data and the range of % seroprevalence. The studies described in S6 table and table 2 are the same studies?

**Authors:** Thank you for thoroughly checking the references. The reference for the study conducted in the U.S. Virgin Islands has been updated (Briskin et al, 2019). The highest seroprevalence was observed in Puerto Rico (not in Trinidad and Tobago) (Artus, 2022). It now reads: “*Seroprevalence varied from 4.1% in U.S. Virgin Islands (2019) (34) to 27.2% in Puerto Rico (2015) (37) (****Table 2****)*.”

Line 288: Please remove an extra %.

**Authors:** The extra ‘%’ has been removed.

Line 296: The S9 table should be italic.

**Authors:** The correction has been made.

Figure 3 legend: Please add the figure legend for white box and the year of the publication should be 2000-2023 or 2000-2022.

**Authors:** The legend for the white box (‘*not reported*’) has been added. The period has been corrected to 2000-2022.

Line 353-356: Please add the references for the data of Saint Lucia.

**Authors:** The two references have been added (Wood et al, 2014 and Cherry et al, 2020).

S5 table: the column of “Impact of PH intervention”, what is PH stand for?

**Authors:** PH stands for public health. The acronym has been replaced by ‘public health’.

S5 and S6 table: It will be useful if the reference of each publication can be added.

**Authors:** We agree that this facilitates reading and comparison across the two supplementary tables and the main text. The references have been added.

**Reviewer #4:** The analysis matches the plan. Most of the results were clearly and completely presented. A few comments in Table 1. There is a missing unit of incidence in Table 1, which is to ensure that the values were adjusted by year and population in the study area. It is not clear to me what you mean by "Not population representative" in the footnote of Table 1. I suggest describing more either in the text or in the footnote. Lastly, although the "Lab confirmed rate" is useful information, the description of tests (used to confirm cases) is also important for demonstrating the case report accuracy in each study. I suggest adding the methodology used for case confirmation in Table 1 and/or in the text.

The incidence unit (100,000 cases per people) was added to the table footnote.

The notation ‘^e^*Not population representative’* was replaced by ‘^f^*Does not represent the totality of cases in Guadeloupe during the study period*’ as the two publications with that notation included cases reported only by one of the two hospitals in Guadeloupe and therefore do not represent the totality of reported cases.

The criteria used to report lab-confirmed cases is presented in *S7 Table*. This information was added to the table footnote.

**Conclusions**

**Reviewer #2:**

There are some analyses made in the results section after the description of that evidence that are not fully supported by the findings (for example, 319-328 (trend increase in incidence when the 2011 numbers were lower than the 2003 and 2004)

**Authors:** The ‘increasing trend’ statement was based on case reports between 2000 and 2004 (from 20 to 103). If taking into account only the number of cases, and not the rate of confirmed cases among suspected, in 2011 there was an increase compared to 2003/2004 (from 62,103 to 126, respectively).

To improve clarity, the following changes were made: “*In 2000, 20 confirmed cases were reported (number of suspected cases not reported) (25), followed by 62 (27.9% of all suspected cases) in 2003 and 103 (38.7% of all suspected cases) in 2004 (26). These results suggested an increasing trend in the number of cases in the early 2000s*.”

and 339-349 (they suggest misdiagnosis without considering coinfection)).

**Authors:** Co-infection could also justify why some cases reported as dengue had positive leptospirosis IgM. According to the study being discussed in this highlighted section (Lindo et al, 2013), 0.2% of all samples were positive for the three infections being investigated (dengue, leptospirosis and malaria), coinfection between dengue and leptospirosis was detected in 1.9% of all samples. To address this topic, the following changes were made:

“*In this study, 6.5% of all samples tested were positive (ELISA IgM Ab) for leptospirosis and 1.9% were positive for both dengue and leptospirosis*”.

The authors never make references to the limitations of the studies they are reporting on, and present the results of these studies as generalizable facts.

**Authors:** Thank you for highlighting this very important limitation of this manuscript. To address this issue, we added the following paragraph into the discussion section (lines 449-451): “*Additionally, the included publications varied substantially, reflecting differences in sampling design, study populations, diagnostic methods, and reporting practices, which further limits the comparability of findings and their generalisability*.”

The authors outline the importance of their findings, gaps in knowledge around leptospirosis in the insular Caribbean Region.
Public health relevance is addressed

**Authors:** No changes were suggested.

**Reviewer #3:**

In the discussion, the results are discussed in several aspects including extreme weather associated with leptospirosis epidemiology, leptospirosis cases in rural and urban settings and the number of leptospirosis cases (reported and estimated) in CRICTs. However, some results from data analyses are not discussed such as the risk factors from surveillance-based studies and seroprevalence studies and circulating serogroups presented in CRICTs.
**Authors:** Discussion regarding risk factors and drivers of transmission has been complemented by the following paragraph (lines 414-418):

“*Although the number of studies investigating risk factors associated with leptospirosis transmission was limited, both surveillance-based and seroprevalence studies indicated a higher risk associated with occupation and animal exposure (26, 28, 33-35), which might suggest that living in rural areas and work environment context were relevant to leptospirosis transmission in the CR*. “

Discussion regarding serovars were included (lines 437-447):

*“Better understanding circulating serogroups in the region and across countries can help identifying relevant reservoirs and transmission pathways, and may have public health implications (e.g., potential association with disease severity, definition of a regional MAT panel and targeted serovars in vaccines) (53-55). Unfortunately, serogroup data were available for fewer than half of the included publications and were restricted to a small number of CRICTs (25-27, 33-35, 37), with repeated reporting occurring only in Puerto Rico and Guadeloupe. Considerable heterogeneity in the composition of the MAT panel further limited comparability, as differences in* *serovar selection for the MAT panel influence the likelihood of detecting specific serogroups and may underestimate locally circulating strains. Moreover, reliance on ELISA-based screening in routine surveillance means that serogroup information was typically derived from a subset of cases who underwent confirmatory testing, potentially biasing results towards more severe presentations.”*

The limitations of the study are clearly described. The conclusions are supported by results and the public health relevance is addressed.

**Authors:** No changes were suggested.

**Reviewer #4:** Conclusions supported by the data. The limitations are clearly discussed. And the public health relevance is addressed.

**Authors:** No changes were suggested.

**Editorial and Data Presentation Modifications?**

**Reviewer #2:**

Given that some of the studies used specific populations (pregnant women, hospitalized patients with clinical suspicion of leptospirosis) and sample sizes (representative?), and that some of the tests used do not constitute diagnostic confirmation, the authors should be careful how they phrase their analysis to make their results represent the whole incidence or hospitalization rate for a CRICT.

**Authors:** Thank you for highlighting this very important limitation of this manuscript. To address this issue, we added the following paragraph into the discussion section (lines 449-451): “*Additionally, included publications varied substantially, reflecting differences in sampling design, study populations, diagnostic methods, and reporting practices, which further limits the comparability of findings and their generalisability*.”

There seems to be a few discussion elements in the results section. For example, in the section providing data on the findings in each CRICT, the authors follow up the report of what was found with an interpretation of those results. Such interpretations are usually reserved for the discussion section.

**Authors:** These sentences with interpretation of results were removed from this section.

Specific comments:
95-97: The author's statement about 16.4% of global outbreaks taking place in the CR is not supported by the study used as a reference.

**Authors:** Thank you for checking the references. Our statement *“A study investigating leptospirosis outbreaks between 1970 and 2012 identified that 16.4% of global outbreaks were reported in the CR”* is based on the cited publication Munoz-Zanzi et al, 2020.

In the cited study, 318 outbreaks were identified worldwide. Of those, 52 were in the Caribbean. In other words, 52 of the 318 outbreaks were from the Caribbean, which is 16.4%. (The information can be found under ‘Regional distribution’, page 3.

No changes were made.

170-174: The author mentions 6 groups but only describes 5

**Authors:** The correct number is five categories, and it has been corrected.

215: I think a clear definition of “routine surveillance” might help the reader interpret the authors' findings.

**Authors:** Both ‘routine surveillance systems-based studies’ and ‘seroprevalence studies’ were defined in the Methods section (lines 156-159).

“*(i) routine surveillance systems-based studies, which reported cases or population-level incidence obtained from existing surveillance systems (e.g., clinical and laboratory reporting, or systematic notification of suspected cases), and (ii) seroprevalence studies, which reported seroprevalence based on the detection of anti-Leptospira antibodies (Ab).*”

319-328: Can you really talk about a trend increase in incidence when the 2011 numbers were lower than the 2003 and 2004 numbers?

**Authors:** The ‘increasing trend’ statement was based on case reports between 2000 and 2004 (from 20 to 103). If taking into account only the number of cases, and not the rate of confirmed cases among suspected, in 2011 there was an increase compared to 2003/2004 (from 62,103 to 126, respectively).

To improve clarity, the following changes were made: “*In 2000, 20 confirmed cases were reported (number of suspected cases not reported) (25), followed by 62 (27.9% of all suspected cases) in 2003 and 103 (38.7% of all suspected cases) in 2004 (26). These results suggested an increasing trend in the number of cases in the early 2000s*.”

339-349: Can you really reach that conclusion, considering the possibility of coinfections or the evidence that Dengue virus-infected patients can generate false positives in Leptospira spp. MAT?

**Authors:** We agree that the challenges regarding leptospirosis misdiagnosis and underreporting are much more complex than the clinical overlap with dengue infection. Additionally, it was also pointed out that this type of conclusion was misplaced in the results section. To address the challenges without drawing conclusions, the following changes were made: “*In this study, 6.5% of all samples tested were positive (ELISA IgM Ab) for leptospirosis and 1.9% were positive for both dengue and leptospirosis.*”

347-349: You should probably include the month of Hurricane Fiona to make that information more impactful to the reader

**Authors:** The complete landfall date has been added.

366: Please specify the criteria you are alluding to?

**Authors:** The following changes were made to improve clarity *“(…) 1% of active sugarcane fieldworkers presented laboratory criteria (i.e., positive immunoglobulin M (IgM) Enzyme-linked Immunosorbent Assay (ELISA) suggestive of acute leptospirosis*.”

424-425: Can study designs have impacted hospitalization rates?

**Authors:** Yes. This is an important aspect that could have impacted hospitalisation rates. This was added to the examples provided in the discussion.

*“Additionally, hospitalisation rate can be impacted by several contextual aspects (e.g., access to care, availability of outpatient services and study design), further hindering comparisons across countries and territories, especially when the data are scarce.”*

**Summary and General Comments**

**Reviewer #2**: This is a relevant study that puts into evidence that leptospirosis is not only a neglected but also forgotten tropical disease. Its methodological structure is solid, the paper is well written, and the authors' findings are supported by what was reported. The authors need to be more critical of the evidence they are reporting on, especially when generalize one study's findings as evidence at a national level.

Thank you again for highlighting this very important limitation of this manuscript. As previously described, were added the following to the discussion, to highlight the limitation of comparison between studies and generalisation of results (lines 449-451): “*Additionally, the quality of the included publications varied substantially, reflecting differences in sampling design, study populations, diagnostic methods, and reporting practices, which further limits the comparability of findings and their generalisability*.”

**Reviewer #3:** The study provides valuable update data of leptospirosis epidemiology in CRICTs although the relevance studies in this region are limited. The results reveal knowledge gaps, offering opportunities to strengthen research and surveillance systems in this region.

**Authors:** No changes were suggested.
